# Increased taurine in pre-weaned juvenile mdx mice greatly reduces the acute onset of myofibre necrosis and dystropathology and prevents inflammation

**DOI:** 10.1371/currents.md.77be6ec30e8caf19529a00417614a072

**Published:** 2016-04-29

**Authors:** Jessica R. Terrill, Miranda D Grounds, Peter G. Arthur

**Affiliations:** The University of Western Australia; School of Anatomy and Human Biology, the University of Western Australia, Perth, Australia.; School of Chemistry and Biochemistry, The University of Western Australia, Perth, Western Australia

## Abstract

Background: The mdx mouse model for the fatal muscle wasting disease Duchenne Muscular Dystrophy (DMD) shows a very mild pathology once growth has ceased, with low levels of myofibre necrosis in adults. However, from about 3 weeks of post-natal age, muscles of juvenile mdx mice undergo an acute bout of severe necrosis and inflammation: this subsequently decreases and stabilises to lower adult levels by about 6 weeks of age. Prior to the onset of this severe dystropathology, we have shown that mdx mice are deficient in the amino acid taurine (potentially due to weaning), and we propose that this exacerbates myofibre necrosis and inflammation in juvenile mdx mice. Objectives: The purpose of this study was to increase taurine availability to pre-weaned juvenile mdx mice (from 14 days of age), to evaluate the impact on levels of myofibre necrosis and inflammation (at 22 days) during the acute period of severe dystropathology. Results: Untreated 22 day old mdx muscle was not deficient in taurine, with similar levels to normal C57 control muscle. However taurine treatment, which increased the taurine content of young dystrophic muscle (by 40%), greatly reduced myofibre necrosis (by 75%) and prevented significant increases in 3 markers of inflammation. Conclusion: Taurine was very effective at preventing the acute phase of muscle damage that normally results in myofibre necrosis and inflammation in juvenile mdx mice, supporting continued research into the use of taurine as a therapeutic intervention for protecting growing muscles of young DMD boys

## Introduction

Duchenne Muscular Dystrophy (DMD) is a lethal, X-chromosome linked muscle disease affecting about 1 in 3500-6000 boys worldwide (Reviewed in 1 ^,^ 2). DMD is caused by the loss of functional dystrophin protein in muscle that results in increased necrosis and inflammation after muscle contraction^3^ ^4^^,^ 5 ^,^ 6. Repeated cycles of widespread myofibre necrosis and progressive failure of regeneration (with replacement of myofibres by fatty and fibrous connective tissue) lead to the loss of muscle mass and function in DMD boys, with premature death often due to respiratory or cardiac failure (Reviewed in 1 ^,^ 7). There is no cure for DMD and the standard drug treatment for DMD, corticosteroids, are limited in their efficacy and are associated with severe side effects^8^. Consequently, there is considerable interest in pharmacological interventions and nutritional supplementation as potential therapies to reduce disease severity (reviewed in 9 ^,^ 10 ^,^ 11).

Much DMD research utilises the adult mdx mouse model to test potential therapies. Whilst the adult mdx mouse has a very mild pathology, the juvenile mdx mouse undergoes an acute onset of severe myofibre necrosis, associated with many inflammatory cells (and subsequent myogenesis and new muscle formation) between 3 and 4 weeks of age^12^^,^ 13. Therefore, the capacity of a therapy to prevent necrosis of juvenile mdx muscles would be a rigorous test of the efficacy of a potential clinical treatment, especially considering that DMD first manifests in young growing boys.

We and others have shown that the amino acid taurine decreases inflammation and improves muscle strength in adult mdx mice^14^^,^ 15 ^,^ 16 ^,^ 17. Taurine is found in many tissues and is considered important for the function of skeletal muscle, where it modulates ion channel function, membrane stability and calcium homeostasis, as well as having anti-inflammatory and antioxidant properties^18^^,^ 19 ^,^ 20 ^,^ 21 ^,^ 22 ^,^ 23. The effect of taurine treatment on severe myofibre necrosis in juvenile mdx mice has not been investigated. One reason for this is that juvenile pre-weaned mice are not routinely available from some commercial suppliers of mdx mice: thus many pre-clinical studies are limited to interventions using older mice.

We have shown that prior to the onset of pathology (18 days), mdx mice are deficient in taurine^24^. Mouse milk is very rich source of taurine, being the most abundant amino acid in mouse milk^25^. From about 10-17 days of age mouse pups begin eating solid food, and milk production of the mother dramatically drops between 16 and 21 days^26^^,^ 27. Since standard mouse chow is almost devoid of taurine, we propose that weaning (and subsequent associated drop in taurine ingestion) leads to a taurine deficiency in juvenile mdx mice, which initiates muscle necrosis.

To investigate the proposed contribution of taurine deficiency to susceptibility of young growing mdx muscles to necrosis, juvenile mdx mice were given access to taurine enriched chow from 14 days until sampling at 22 days (after the initiation of myofibre necrosis). Quadriceps muscles from untreated and taurine treated mdx mice, and untreated control normal C57Bl/10Scsn (C57) mice were analysed for taurine content, myofibre necrosis and markers of inflammation (neutrophil elastase, myeloperoxidase (MPO) and the pro-inflammatory cytokine tumour necrosis factor [TNF]). Taurine treatment increased the taurine content of mdx quadriceps muscles, and resulted in a striking decrease in myofibre necrosis and inflammation, providing further support for taurine as a potential intervention for growing DMD boys.

## Methods

All reagents used were obtained from Sigma Aldrich unless otherwise specified.

**Animal procedures** All experiments were carried out on dystrophic mdx (C57Bl/10ScSnmdx/mdx) and non-dystrophic control C57 (C57Bl/10ScSn) mice (the parental strain for mdx). All mice were obtained from the Animal Resource Centre, Murdoch, Western Australia. Mice were maintained at the University of Western Australia on a 12-h light/dark cycle, under standard conditions, with free access to food and drinking water. All animal experiments were conducted in strict accordance with the guidelines of the National Health and Medical Research Council Code of practice for the care and use of animals for scientific purposes (2004), and the Animal Welfare act of Western Australia (2002), and were approved by the Animal Ethics committee at the University of Western Australia.

From 14 days of age, mice had access to soft chow, with taurine treated mice receiving 4% taurine in their chow. Each group contained n=8 pups, with approximately equal proportions of male and female pups (~50:50). There was no observable difference between male and female mice. All mice were sacrificed at 22 days by cervical dislocation while under terminal anesthesia (2%v/v Attane isoflurane Bomac Australia). Quadriceps muscles were collected and immediately snap frozen in liquid nitrogen for biochemical analysis, or prepared for histology by immersing in 4% paraformaldehyde before possessing for paraffin histology.

**Taurine content of muscle** Taurine in muscle was measured using reverse phase high performance liquid chromatography (HPLC) as previously described^28^. Frozen quadriceps muscles were crushed using a mortar and pestle under liquid nitrogen and homogenized in 25 times 5% TCA, and plasma samples were precipitated by addition of 10 times by weight of 5% TCA. After centrifugation, supernatants were removed and stored at -80°C before analysis. Analytes were separated using HPLC with fluorescent detection, with pre-column derivitisation with o-phthalaldehyde (OPA) and 2-mercaptoethanol (2ME). Supernatants were mixed with iodoacetamide, dissolved in 5% TCA, to a final concentration of 25 mM. An internal standard, o-phospho-dl-serine, dissolved in 5% TCA was added to a final concentration of 5 mM. Sodium borate was used to adjust the pH to 9. Samples were mixed on a sample loop with a derivatising solution containing 40 mM OPA and 160 mM 2ME in 100 mM sodium borate, pH 12, for 30 seconds before injection onto the column. Separation was achieved with a C18 column (5 µl, 4.6 x 150 mm, Phenomenex) using a Dionex Ultimate 3000 HPLC system. Mobile phase A consisted of 50 mM potassium phosphate buffer, methanol and tetrahydrofuran (94:3:3). Mobile phase B consisted of 90% methanol, with a gradient increase in B from 0 to 25%. Fluorescence was set at 360 nm and 455 nm for excitation and emission respectively. The protein content of muscle samples were quantified by solubilising the pellet in 0.5 M sodium hydroxide, before incubation at 80°C for 15 minutes. Once fully dissolved, protein concentrations of supernatants were quantified using a Bradford protein assay (Bio-Rad).

**Myofibre necrosis** Histological analysis was completed as per the TREAT-NMD recommended standard protocol “Histological measurements of dystrophic muscle - M.1.2_007” http://www.treat-nmd.eu/downloads/file/sops/dmd/MDX/DMD_M.1.2.007.pdf

Transverse muscle sections (5 μm) were cut through the mid-region of each quadriceps muscle on a Leica microtome, and sections were stained with Haematoxylin and Eosin (H&E) for morphological analysis.

Myofibre necrosis was identified as areas of myofibres with fragmented sarcoplasm and/or increased inflammatory cell infiltration, and was measured using non-overlapping tiled images of transverse muscle sections that provided a picture of the entire muscle cross section. Tiled digital images were captured at x10 magniﬁcation using a Nikon Eclipse Ti inverter microscope equipped with Nikon DS-Fi2 camera (Nikon Corporation). Analysis was performed blind, and areas of necrosis drawn manually by the researcher using Image Pro Plus 4.5.1 software.

**Neutrophil elastase and TNF content of muscle** Frozen quadriceps muscles were crushed using a mortar and pestle under liquid nitrogen and homogenized in ice-cold 1% NP40, 1 mM EDTA in phosphate buffered saline (PBS), supplemented with complete EDTA free protease inhibitor tablets and PhosSTOP phosphatase inhibitor tablets (Roche), and centrifuged. The protein concentration of supernatants was quantitated using the Detergent Compatible (DC) protein assay (Bio-Rad). Samples were resolved on 4-15% SDS-PAGE TGX gels (Bio-Rad) and transferred onto nitrocellulose membrane using a Trans Turbo Blot system (Bio-Rad). Immuno-blotting was performed on the same membrane with antibodies to neutrophil elastase (ab68672, Abcam), TNF (AB2148P, Chemicon), and glyceraldehyde 3-phosphate dehydrogenase (GAP, 14C10, Cell Signalling), all dissolved 1:1000 in 5% bovine serum albumin (BSA). HRP-conjugated secondary antibodies were from Thermo Fisher Scientific. Chemiluminescence signal was captured using the ChemiDoc MP Imaging System (Bio-Rad). Resultant images were quantified using ImageJ software^29^. Glyceraldehyde 3-phosphate dehydrogenase loading controls were immunoblotted on the same membrane as immunoblotted proteins, and signals for neutrophil elastase and TNF were standardised to this loading control.

**Myeloperoxidase (MPO) content of muscle** The enzyme MPO catalyses the production of hypochlorous acid from hydrogen peroxide and chloride^30^ and hypochlorous acid reacts with 2-[6-(4-aminophenoxy)-3-oxo-3H-xanthen-9-yl]benzoic acid (APF) to form the highly fluorescent compound fluorescein, that is measured in this method, as per [28]. Briefly, frozen quadriceps muscles were ground using a mortar and pestle under liquid nitrogen and homogenised in 0.5% hexadecyltrimethylammonium bromide in PBS. Samples were centrifuged and supernatants diluted in PBS. Human MPO was used as the standard for the assay (Cayman Chemical). Aliquots of each experimental sample or MPO standard was pipetted into a 384 well plate, before the addition of APF working solution (20 µM APF [Cayman Chemical] and 20 µM hydrogen peroxide in PBS) was added. The plate was incubated at room temperature (protected from light) for 30 minutes, with the fluorescence being measured every minute using excitation at 485 nm and emission at 515-530 nm. The rate of change of fluorescence for each sample was compared to that of the standards and results were expressed per mg of protein, quantified using the DC protein assay (Bio-Rad).

**Statistics** Data were analysed using GraphPad Prism software. One-way ANOVA tests with post-hoc (LSD) comparisons were used to identify significant differences between experimental groups. Statistical significance was accepted at p<0.05. All data are presented as mean ± SEM.

## Results

**Fig. 1. Taurine content of C57, untreated mdx and taurine treated mdx quadriceps muscles, from mice aged 22 days:**
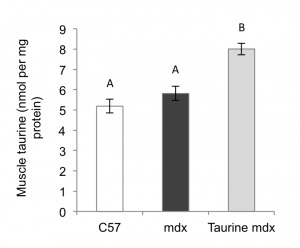
Data are presented as mean ± SEM and n= 8 mice/group. Groups without a common letter are significantly (p<0.05) different.

**Muscle taurine content** There was no significant difference between the taurine content of C57 and untreated mdx muscle at 22 days of age (Fig. 1). Taurine treatment of juvenile mdx mice for 8 days resulted in a 1.4 fold increase in muscle taurine content.

**Fig. 2. Myofibre necrosis in C57, untreated mdx and taurine treated mdx quadriceps muscle, from mice aged 22 days:**
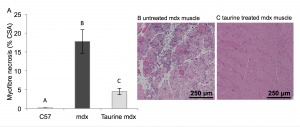
(A) Histological quantification of myofibre necrosis. Data are presented as mean ± SEM of percentage of cross section area (CSA) and n= 8 mice/group. Groups without a common letter are significantly (p<0.05) different. Representative images of myofibre necrosis and histology of H&E stained muscle sections are shown for (B) untreated mdx (C) taurine treated mdx mice.

**Muscle necrosis** Myofibre necrosis was minimal in normal C57 quadriceps muscle, whereas myofibre necrosis was conspicuous and represented about ~17% of the cross-sectional area of the quadriceps in untreated mdx mice aged 22 days (Fig. 2). Taurine treatment of mdx mice significantly reduced (by 4 fold) myofibre necrosis (to ~5%) (Fig. 2).

**Fig. 3. Quantification of inflammation in C57, untreated mdx and taurine treated mdx quadriceps muscles, from mice aged 22 days:**
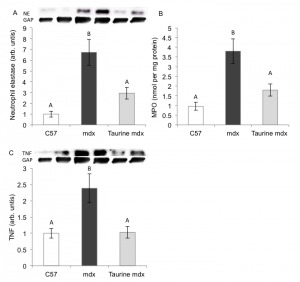
Measurements are of (A) Neutrophil elastase, (B) MPO and (C) TNF. Data are presented as mean ± SEM and n= 8 mice/group. Groups without a common letter are significantly (p<0.05) different. Representative blots are shown of neutrophil elastase, TNF and the loading control glyceraldehyde 3-phosphate dehydrogenase (GAP).

**Muscle Inflammation** Neutrophil accumulation is a hallmark of acute inflammation, and we assessed the incidence of neutrophils by western blotting for the protein neutrophil elastase and by measuring the activity of MPO, an enzyme secreted by inflammatory cells (primarily by neutrophils) that facilitates their antimicrobial activity. Neutrophil elastase and MPO activity were 6.7 and 4 fold higher (respectively) in mdx compared to C57 muscle (Fig. 3). Taurine treatment of mdx mice reduced neutrophil elastase protein and MPO activity by 2.3 and 2 fold, respectively (Fig. 3): these reduced levels were not significantly different to those in normal C57 muscles (Fig. 3).

Protein levels of the pro-inflammatory cytokine TNF measured by western blotting (Fig. 3C) were 2.4 fold higher in mdx compared to C57 muscles. Taurine treatment of mdx mice resulted in a striking 2.4 fold reduction in muscle TNF content (compared with untreated mdx), to the same low levels as in C57 muscles (Fig. 3C).

## Discussion

Taurine administration to juvenile mdx mice from 14 days of age substantially increased muscle taurine content and greatly mitigated the severity of the acute onset of myofibre necrosis and prevented muscle inflammation at 22 days of age. These data are novel and provide strong support for the growing interest in taurine as a potential low cost clinical intervention to protect the muscles of growing DMD boys.

We have previously reported a taurine deficiency in young 18 day old mdx mice, prior to the acute onset of pathology, and this deficiency coincided with the time of weaning of pups from taurine rich milk to taurine poor chow: accordingly, we proposed that weaning (with subsequent drop in taurine ingestion) leads to a taurine deficiency in young mdx mice, which exacerbates muscle necrosis^24^. However, by 22 days taurine levels in mdx mice have recovered to normal control C57 levels. These data are unclear in defining the role of taurine levels in onset of mdx pathology: this may relate to precise timing of changing taurine levels in growing mdx mice between 18-22 days, since the initiation of myofibre necrosis, that occurs just before 22 days, may be intensified by persistent prior taurine deficiency. Many other cellular and molecular factors that change around 21 days in growing mice may also contribute to the timing of acute onset of myonecrosis in dystrophic muscles, and factors to consider also include the impact of growth, increased mechanical activity and loading of juvenile muscles^3^^,^ 31 ^,^ 32.

The early administration of taurine to juvenile mdx mice decreased (compared with untreated mdx mice) and normalised to C57 levels 3 measures of inflammation in muscles at 22 days: neutrophil elastase, MPO activity and TNF content. Neutrophils, key cells involved in acute inflammation, are phagocytes responsible for generation of various pro-inflammatory mediators^33^. After muscle injury, neutrophils rapidly invade the tissue to remove debris, however in doing so can exacerbate muscle damage^34^. A fundamental mechanism of neutrophil mediated damage to muscle is the secretion of MPO, a heme enzyme that oxidises chloride in the presence of hydrogen peroxide to form the potent and damaging oxidant hypochlorous acid (HOCl)^35^. The anti-inflammatory and antioxidant properties of taurine are attributed to its ability to react with hypochlorous acid to form the much less reactive molecule taurine chloramine which itself exerts anti-inflammatory effects such as inhibiting the production of pro-inflammatory cytokines, including TNF^33^.

Activated leucocytes such as neutrophils (as well as many other cell types including macrophages and muscle cells) produce TNF, and therefore TNF content of muscle is elevated after injury^36^. TNF plays several important roles in inflammation such as activation and chemotaxis of leucocytes, and can itself stimulate muscle injury via NF-κB mediated protein degradation^36^^,^ 37. In the current study, these anti-inflammatory properties of taurine may contribute to the decreased inflammation in mdx muscle observed after taurine treatment. In the current study, these anti-inflammatory properties of taurine may contribute to the decreased inflammation observed in mdx muscle after taurine treatment. However the reduced inflammation might simply be a consequence of the reduction in muscle necrosis by taurine (due to another effect such as membrane stabilisation or calcium homeostasis). More experimental research is required to understand the exact mechanism for the benefits of taurine on dystropathology and the protection of juvenile mdx muscle from necrosis and inflammation.

To summarise, taurine treatment was very effective in mitigating the severe bout of necrosis and preventing inflammation in dystrophic muscles of juvenile mdx mice. This is an important observation, since interventions that can protect the vulnerable growing dystrophic myofibres from necrosis could help preserve muscle mass and function in young DMD boys. These novel data support continued preclinical research into the use of taurine as a promising clinical intervention for DMD.

## Competing interests

The authors have declared that no competing interests exist.

## Correspondence

The corresponding author can be contacted at jessica.terrill@uwa.edu.au

## References

[1] Bushby K, Finkel R, Birnkrant DJ, Case LE, Clemens PR, Cripe L, Kaul A, Kinnett K, McDonald C, Pandya S, Poysky J, Shapiro F, Tomezsko J, Constantin C. Diagnosis and management of Duchenne muscular dystrophy, part 1: diagnosis, and pharmacological and psychosocial management. Lancet Neurol. 2010 Jan;9(1):77-93. PubMed PMID:19945913. 19945913 10.1016/S1474-4422(09)70271-6

[2] Emery AE. The muscular dystrophies. Lancet. 2002 Feb 23;359(9307):687-95. PubMed PMID:11879882. 11879882 10.1016/S0140-6736(02)07815-7

[3] Grounds MD. Two-tiered hypotheses for Duchenne muscular dystrophy. Cell Mol Life Sci. 2008 Jun;65(11):1621-5. PubMed PMID:18327663. 18327663 10.1007/s00018-008-7574-8 PMC11131677

[4] Kharraz Y, Guerra J, Pessina P, Serrano AL, Muñoz-Cánoves P. Understanding the process of fibrosis in Duchenne muscular dystrophy. Biomed Res Int. 2014;2014:965631. PubMed PMID:24877152. 24877152 10.1155/2014/965631 PMC4024417

[5] Lapidos KA, Kakkar R, McNally EM. The dystrophin glycoprotein complex: signaling strength and integrity for the sarcolemma. Circ Res. 2004 Apr 30;94(8):1023-31. PubMed PMID:15117830. 15117830 10.1161/01.RES.0000126574.61061.25

[6] Petrof BJ, Shrager JB, Stedman HH, Kelly AM, Sweeney HL. Dystrophin protects the sarcolemma from stresses developed during muscle contraction. Proc Natl Acad Sci U S A. 1993 Apr 15;90(8):3710-4. PubMed PMID:8475120. 8475120 10.1073/pnas.90.8.3710 PMC46371

[7] Biggar WD. Duchenne muscular dystrophy. Pediatr Rev. 2006 Mar;27(3):83-8. PubMed PMID:16510548. 16510548 10.1542/pir.27-3-83

[8] Manzur AY, Kuntzer T, Pike M, Swan A. Glucocorticoid corticosteroids for Duchenne muscular dystrophy. Cochrane Database Syst Rev. 2008 Jan 23;(1):CD003725. PubMed PMID:18254031. 18254031 10.1002/14651858.CD003725.pub3

[9] Radley HG, De Luca A, Lynch GS, Grounds MD. Duchenne muscular dystrophy: focus on pharmaceutical and nutritional interventions. Int J Biochem Cell Biol. 2007;39(3):469-77. PubMed PMID:17137828. 17137828 10.1016/j.biocel.2006.09.009

[10] De Luca A. Pre-clinical drug tests in the mdx mouse as a model of dystrophinopathies: an overview. Acta Myol. 2012 May;31(1):40-7. PubMed PMID:22655516. 22655516 PMC3440805

[11] De Luca A, Pierno S, Camerino DC. Taurine: the appeal of a safe amino acid for skeletal muscle disorders. J Transl Med. 2015 Jul 25;13:243. PubMed PMID:26208967. 26208967 10.1186/s12967-015-0610-1 PMC4513970

[12] McGeachie JK, Grounds MD, Partridge TA, Morgan JE. Age-related changes in replication of myogenic cells in mdx mice: quantitative autoradiographic studies. J Neurol Sci. 1993 Nov;119(2):169-79. PubMed PMID:8277331. 8277331 10.1016/0022-510x(93)90130-q

[13] Grounds MD, Torrisi J. Anti-TNFalpha (Remicade) therapy protects dystrophic skeletal muscle from necrosis. FASEB J. 2004 Apr;18(6):676-82. PubMed PMID:15054089. 15054089 10.1096/fj.03-1024com

[14] Terrill JR, Pinniger GJ, Graves JA, Grounds MD, Arthur PG. Increasing taurine intake and taurine synthesis improves skeletal muscle function in the mdx mouse model for Duchenne Muscular Dystrophy. J Physiol. 2015 Dec 12. PubMed PMID:26659826. 26659826 10.1113/JP271418 PMC4887673

[15] Cozzoli A, Rolland JF, Capogrosso RF, Sblendorio VT, Longo V, Simonetti S, Nico B, De Luca A. Evaluation of potential synergistic action of a combined treatment with alpha-methyl-prednisolone and taurine on the mdx mouse model of Duchenne muscular dystrophy. Neuropathol Appl Neurobiol. 2011 Apr;37(3):243-56. PubMed PMID:20618838. 20618838 10.1111/j.1365-2990.2010.01106.x

[16] De Luca A, Pierno S, Liantonio A, Cetrone M, Camerino C, Fraysse B, Mirabella M, Servidei S, Rüegg UT, Conte Camerino D. Enhanced dystrophic progression in mdx mice by exercise and beneficial effects of taurine and insulin-like growth factor-1. J Pharmacol Exp Ther. 2003 Jan;304(1):453-63. PubMed PMID:12490622. 12490622 10.1124/jpet.102.041343

[17] De Luca A, Pierno S, Liantonio A, Cetrone M, Camerino C, Simonetti S, Papadia F, Camerino DC. Alteration of excitation-contraction coupling mechanism in extensor digitorum longus muscle fibres of dystrophic mdx mouse and potential efficacy of taurine. Br J Pharmacol. 2001 Mar;132(5):1047-54. PubMed PMID:11226135. 11226135 10.1038/sj.bjp.0703907 PMC1572646

[18] Bakker AJ, Berg HM. Effect of taurine on sarcoplasmic reticulum function and force in skinned fast-twitch skeletal muscle fibres of the rat. J Physiol. 2002 Jan 1;538(Pt 1):185-94. PubMed PMID:11773327. 11773327 10.1113/jphysiol.2001.012872 PMC2290020

[19] Hamilton EJ, Berg HM, Easton CJ, Bakker AJ. The effect of taurine depletion on the contractile properties and fatigue in fast-twitch skeletal muscle of the mouse. Amino Acids. 2006 Oct;31(3):273-8. PubMed PMID:16583307. 16583307 10.1007/s00726-006-0291-4

[20] Huxtable RJ. Physiological actions of taurine. Physiol Rev. 1992 Jan;72(1):101-63. PubMed PMID:1731369. 1731369 10.1152/physrev.1992.72.1.101

[21] Warskulat U, Flögel U, Jacoby C, Hartwig HG, Thewissen M, Merx MW, Molojavyi A, Heller-Stilb B, Schrader J, Häussinger D. Taurine transporter knockout depletes muscle taurine levels and results in severe skeletal muscle impairment but leaves cardiac function uncompromised. FASEB J. 2004 Mar;18(3):577-9. PubMed PMID:14734644. 14734644 10.1096/fj.03-0496fje

[22] Warskulat U, Heller-Stilb B, Oermann E, Zilles K, Haas H, Lang F, Häussinger D. Phenotype of the taurine transporter knockout mouse. Methods Enzymol. 2007;428:439-58. PubMed PMID:17875433. 17875433 10.1016/S0076-6879(07)28025-5

[23] Conte Camerino D, Tricarico D, Pierno S, Desaphy JF, Liantonio A, Pusch M, Burdi R, Camerino C, Fraysse B, De Luca A. Taurine and skeletal muscle disorders. Neurochem Res. 2004 Jan;29(1):135-42. PubMed PMID:14992272. 14992272 10.1023/b:nere.0000010442.89826.9c

[24] Terrill JR, Grounds MD, Arthur PG. Taurine deficiency, synthesis and transport in the mdx mouse model for Duchenne Muscular Dystrophy. Int J Biochem Cell Biol. 2015 Sep;66:141-8. PubMed PMID:26239309. 26239309 10.1016/j.biocel.2015.07.016

[25] Rassin DK, Sturman JA, Guall GE. Taurine and other free amino acids in milk of man and other mammals. Early Hum Dev. 1978 Apr;2(1):1-13. PubMed PMID:102507. 102507 10.1016/0378-3782(78)90048-8

[26] Latham N, Mason G. From house mouse to mouse house: the behavioural biology of free-living Mus musculus and its implications in the laboratory. Applied Animal Behaviour Science. 2004;86(3-4):261-89.

[27] Jara-Almonte M, White JM. Milk production in laboratory mice. J Dairy Sci. 1972 Oct;55(10):1502-5. PubMed PMID:5077616. 5077616 10.3168/jds.S0022-0302(72)85703-5

[28] Terrill JR, Boyatzis A, Grounds MD, Arthur PG. Treatment with the cysteine precursor l-2-oxothiazolidine-4-carboxylate (OTC) implicates taurine deficiency in severity of dystropathology in mdx mice. Int J Biochem Cell Biol. 2013 Sep;45(9):2097-108. PubMed PMID:23892094. 23892094 10.1016/j.biocel.2013.07.009

[29] Schneider CA, Rasband WS, Eliceiri KW. NIH Image to ImageJ: 25 years of image analysis. Nat Methods. 2012 Jul;9(7):671-5. PubMed PMID:22930834. 22930834 10.1038/nmeth.2089 PMC5554542

[30] Winterbourn CC, Kettle AJ. Biomarkers of myeloperoxidase-derived hypochlorous acid. Free Radic Biol Med. 2000 Sep 1;29(5):403-9. PubMed PMID:11020661. 11020661 10.1016/s0891-5849(00)00204-5

[31] Grounds M, Shavlakadze T. Impact of growth on properties of sarcolemma of skeletal myofibres: Clinical and scientific implications. Bioessays. 2011;33:458-68. 10.1002/bies.201000136 21500235

[32] Allen DG, Whitehead NP, Froehner SC. Absence of Dystrophin Disrupts Skeletal Muscle Signaling: Roles of Ca2+, Reactive Oxygen Species, and Nitric Oxide in the Development of Muscular Dystrophy. Physiol Rev. 2016 Jan;96(1):253-305. PubMed PMID:26676145. 26676145 10.1152/physrev.00007.2015 PMC4698395

[33] Marcinkiewicz J, Kontny E. Taurine and inflammatory diseases. Amino Acids. 2014 Jan;46(1):7-20. PubMed PMID:22810731. 22810731 10.1007/s00726-012-1361-4 PMC3894431

[34] Tidball JG. Inflammatory processes in muscle injury and repair. Am J Physiol Regul Integr Comp Physiol. 2005 Feb;288(2):R345-53. PubMed PMID:15637171. 15637171 10.1152/ajpregu.00454.2004

[35] Winterbourn CC. Biological reactivity and biomarkers of the neutrophil oxidant, hypochlorous acid. Toxicology. 2002 Dec 27;181-182:223-7. PubMed PMID:12505315. 12505315 10.1016/s0300-483x(02)00286-x

[36] Collins RA, Grounds MD. The role of tumor necrosis factor-alpha (TNF-alpha) in skeletal muscle regeneration. Studies in TNF-alpha(-/-) and TNF-alpha(-/-)/LT-alpha(-/-) mice. J Histochem Cytochem. 2001 Aug;49(8):989-1001. PubMed PMID:11457927. 11457927 10.1177/002215540104900807

[37] Li YP, Reid MB. NF-kappaB mediates the protein loss induced by TNF-alpha in differentiated skeletal muscle myotubes. Am J Physiol Regul Integr Comp Physiol. 2000 Oct;279(4):R1165-70. PubMed PMID:11003979. 11003979 10.1152/ajpregu.2000.279.4.R1165

